# Authentication Mechanisms in the Internet of Things: A Comparative Analysis Across RFID, Smart Grids, Vehicular Networks, and Smart Homes

**DOI:** 10.3390/s26185893

**Published:** 2026-09-17

**Authors:** Supraja Ayyamgari, Bala Yashwanth Reddy Thumma, Nivedan Suresh, Charan Thumma, Abhignan Srivatsava Sribhashyam, Bhargavi Konda, Mounica Yenugula, Vinay Kumar Kasula

**Affiliations:** 1 Department of Information Technology, University of the Cumberlands, Williamsburg, KY 40769, USA; sayyamgari32587@ucumberlands.edu (S.A.); bkonda19519@ucumberlands.edu (B.K.); myenugula3188@ucumberlands.edu (M.Y.); 2 Independent Researcher, Ashburn, VA 20148, USA; balayrthumma@gmail.com; 3 Independent Researcher, Austin, TX 78759, USA; nivedhan4a8@gmail.com; 4 Independent Researcher, Herndon, VA 20171, USA; charan.thumma@gmail.com; 5 Independent Researcher, Lakeville, MN 55044, USA; sribhashyamabhignan@gmail.com

**Keywords:** IoT authentication, authentication protocols, security risks, resource limitations, scalability, cyber threats, RFID systems, smart grids, vehicular networks, smart homes, lightweight cryptography, federated identity, OAuth, entropy, post-quantum authentication

## Abstract

The Internet of Things (IoT) is envisioned to link billions of diverse devices together. To safeguard privacy of user data, ensure authenticity of the data communicated between these devices, and guarantee their availability, robust authentication is needed. On the other hand, authentication techniques face several security challenges because IoT devices are resource-constrained (memory, battery, processor, etc.). To elaborate on this, we perform an extensive survey of authentication mechanisms in IoT, addressing the resource-constrained nature of devices, heterogeneity of networks, and prospective security challenges. We focus on five major classes of authentication protocols—namely, password-based, MAC-based, public identity-based, token-based, and biometrics-based protocols—and compare them based on communication and computation overhead, energy efficiency, scalability, and security. We also highlight the applicability of authentication mechanisms to four popular IoT platforms: RFID systems, smart grids, the Internet of Vehicles (IoV), and smart homes. Finally, we survey trending solutions such as implicit authentication using biometrics, blockchain-based identity management, and trusted computing. Beyond the qualitative comparison, this updated survey presents a formal, PRISMA-style review process including inclusion and exclusion criteria; a quantitative/information theoretic comparison of authentication classes (using Shannon entropy and confusion- matrix-derived metrics, the false acceptance rate, false rejection rate, equal error rate, and receiver operating characteristic reasoning); and discussion of higher-layer auth architectures including federated identity management, single sign-on (SSO), and OAuth/OpenID Connect that enable device-level protocols. Existing systematic reviews and auth schemes published since 2023 are also included to supplement previously compared device-centric works. Our analysis shows that token-based authentication currently offers the most balanced trade-off among security, scalability, and energy overhead for general-purpose IoT deployments, while biometric and implicit biometric schemes provide the strongest resistance to impersonation at higher computational cost and public-identity (asymmetric) schemes scale well but remain impractical for the most resource-constrained endpoints. In summary and to guide future work directly, the survey also outlines four promising directions towards which the community should further research: (i) hybrid schemes based on lightweight cryptography and behavioral or physiological biometrics; (ii) lightweight post-quantum authentication schemes, resistant to quantum computer attacks and deployable on constrained devices; (iii) continuous authentication and anomaly detection based on machine learning techniques; and (iv) federated and decentralized (blockchain-enabled) identity management solutions towards the minimization of single points of failure among heterogeneous IoT areas.

## 1. Introduction

### 1.1. Evolution of the Internet of Things Concept

The Internet of Things (IoT) concept was first introduced in the 1990s by Professor Kevin Ashton of the Massachusetts Institute of Technology (MIT). In 1999, MIT established the Auto-ID Center, co-founded by Professor Ashton, which defined the IoT as a network connecting objects to the Internet through technologies like radio-frequency identification (RFID) [1]. In 2005, the International Telecommunication Union (ITU) expanded on this definition in the ITU Internet Report 2005: The Internet of Things, describing the IoT as a new communication paradigm enabling information exchange between people and objects or among objects themselves [2]. In 2017, the national GB/T 33745-2017 [3] standard, Terminology for the Internet of Things, provided a more detailed definition: IoT is an intelligent service system that connects objects, people, systems, and information resources through sensing devices and agreed-upon protocols, enabling data processing and response in both physical and virtual worlds [4]. Here, “objects” refer to physical entities.

As IoT has matured, its scope has expanded, and its system typically comprises three entities: users, IoT devices, and IoT services [5,6].

### 1.2. Current Status and Trends in IoT Development

The Internet of Things has advanced significantly in recent years due to breakthroughs in information and communication technology. IoT innovation has been fueled by technologies like Long-Range (LoRa) communication inside the Low-Power Wide-Area (LPWA) network ecosystem, Enhanced Machine-Type Communication (eMTC), and Narrowband IoT (NB-IoT). A golden age of development with a wide range of applications and quickly growing sectors has also been brought about by the rapid developments in big data and artificial intelligence, which have expedited the rise of the IoT. Public health, smart grids, intelligent transportation, waste management, smart homes, smart cities, agriculture, and energy management are just a few of the industries that use IoT devices and are expanding at an exponential rate. According to a Cisco projection based on IDC (International Data Corporation) research, there will be 28.6 billion IoT devices in use worldwide by 2022 [5]. According to reports, by 2025, the value of the IoT might reach trillions of dollars annually [5]. A GSMA Intelligence report projects that, assuming a 15% annual growth rate, there will be 25.2 billion IoT device connections worldwide by 2025 [7]. By 2025, there will be 5.38 billion IoT connections in India [7]. Furthermore, 61% of firms currently use IoT platforms in their operations, with the IT and telecoms sectors leading the way, at 71%, according to Kaspersky’s 2020 report “With Superpower Comes Super Responsibility: Benefits and Challenges of IoT in Business” [8]. This growth in deployment has been accompanied by a corresponding rise in security investment and threat activity, with industry analysts projecting billions of dollars in annual IoT security spending and threat-intelligence reporting documenting a parallel increase in malware targeting connected devices [9,10,11].

#### 1.2.1. IoT Drives Emerging Applications Like Smart Cities

The rapid development of the IoT has led to new uses in wearable technology, smart cities, and smart homes. According to Kaspersky’s analysis, the IoT application domain with the greatest rate of growth is smart cities [8]. By utilizing automated sensing, data gathering, and intelligent control to maximize traffic management, transit management, and video monitoring, the IoT enables smart cities. Numerous Indian cities are implementing “smart city” projects in fields like environmental preservation, healthcare, public safety, transportation, and monitoring of subterranean infrastructure.

#### 1.2.2. IoT Enables Digital Transformation in Traditional Industries

IoT enables digital transformation in sectors like manufacturing, logistics, transportation, healthcare, and agriculture. For example, IoT makes it possible to track transport trucks and monitor cargo in real time, which improves productivity and streamlines logistical procedures. By connecting sensors to machinery, Industrial IoT (IIoT) enables data gathering and analysis using big data technologies to enhance intelligent decision-making and boost productivity in production.

### 1.3. Review Methodology, Search Strategy, and Inclusion/Exclusion Criteria

The candidate studies were gathered from IEEE Xplore, ACM Digital Library, ScienceDirect, SpringerLink, and Scopus using keyword combinations from “IoT authentication”, “lightweight cryptography”, “RFID authentication”, “smart grid authentication”, “Internet of Vehicles authentication”, “smart home authentication”, “federated identity”, “OAuth IoT”, and “post-quantum IoT” from 2012–2026, prioritizing literature published in 2023–2026 where possible so that the discussion of contemporary solutions is focused on state-of-the-art protocols rather than ones that have been surveyed extensively in previous works [12,13,14].

We included (i) peer-reviewed journal articles, peer-reviewed conference papers, and systematic reviews; (ii) studies that proposed, presented evaluation results for or systematically reviewed an authentication mechanism or identity-management technique that could be applied to resource-constrained or heterogeneous IoT deployments; and (iii) studies that reported at least one data point or metric pertaining to computational overhead, communication overhead, energy consumption, scalability, or formal/informal security analysis. We excluded (i) studies that did not focus on identity verification or access control (e.g., papers focused exclusively on networking protocols or routing); (ii) duplicate or otherwise outdated/preprint versions in favor of the newest, peer-reviewed version when faced with multiple versions of the same study; (iii) non-peer-reviewed preprints if a peer-reviewed version of the study was published later; and (iv) studies that were only published as abstracts with limited methodological detail. Title/abstract screening was followed by full-text screening to determine eligibility, as performed in [12,13]—reviews following the PRISMA style—that also surveyed IoT authentication protocols.

Making and adhering to this protocol does not change the scope of the survey—which remains five authentication categories (password-based, MAC-based, public identity-based, token-based, and biometric) across four application domains (RFID, smart grids, IoV, and smart homes)—but it does explain how certain schemes were chosen for comparison while also giving readers a reproducible strategy from which the claims made by Section 3.2.6, Section 3.2.7, Section 3.2.8 and Section 3.2.9 and Section 4.6 were derived rather than a free-text literature walk-through.

## 2. IoT Authentication Security Issues and Challenges

New security threats associated with the use of emerging technologies have also emerged as a result of the rapid expansion of the Internet of Things (IoT) industry. Attacks on IoT networks have the potential to cause system paralysis, which might have detrimental effects on human health, life and property safety, and national security. IoT security events have been increasingly frequent in recent years, and their effects on data security, user privacy, and network infrastructure as a whole have become more noticeable. Twenty percent of firms have had at least one IoT attack in the last three years, according to a Gartner report [9,10,11,15,16].

### 2.1. IoT Security Risk Points

The security challenges of IoT are more complicated than those of conventional Internet systems. In general, three layers make up the IoT network architecture: the application service layer, the network transmission layer, and the terminal access layer. Application services, communication networks, and terminals are the three categories of security concerns.

- Hazards to Terminal Security: These mostly appear in data leakage, network communication structure security, device physical security, and security flaws [1,2]. An IoT device may join a botnet, like Mirai, if it is compromised or taken over by hackers [4,5]. Software-defined networking (SDN) approaches have been proposed to detect and mitigate the resulting distributed denial-of-service (DDoS) traffic at the network level [16].
- Communication Network Risks: Unauthorized access, network breaches, and the security of wireless data transmission links are the key concerns in communication networks.
- Application Service Risks: Due to their diversified application logic and massive data storage, application services are susceptible to attacks. Hackers can take advantage of flaws in the foundational environment and components of the system.

Another Gartner analysis estimates that in 2018, IoT security breaches cost the world $1.5 billion [17,18]. Costs for fault correction, security failures, and recalls are predicted to make up over half of all security protection costs for different businesses by 2022. A number of well-known IoT security problems have occurred in recent years:

- October 2016: Attackers used malware to take over numerous cameras on the US West Coast by taking advantage of security flaws. They caused extensive communication disruptions by launching a Distributed Denial of Service (DDoS) attack against domain name system (DNS) servers.
- 2017: More than 1000 Lexmark printers without password protection were made public online. Attackers could carry out tasks like installing backdoors or taking over print jobs on these devices, which were connected to government organizations in different nations.
- 2018: Due to flaws that allowed unwanted access, a Spanish manufacturer of IoT equipment made its electric car-charging system available online. Attackers might upgrade firmware or steal confidential information.
- December 2019: A home’s smart camera in Mississippi, USA, was compromised. User security and privacy were seriously jeopardized, since the attackers could communicate with the inhabitants and monitor their activities.

### 2.2. IoT Authentication Process

The first and most important step in granting users and devices access to an IoT system is authentication. It guarantees that information resources can only be accessed by authorized entities, and a user-to-user authentication strategy usually consists of several steps.

1. Phase of Initialization: The cloud server (S) securely stores the unique identification (ID_i_) and initial secret key (k_i_) of each user (U_i_). Identity verification and subsequent authentication are conducted using these credentials.
2. Steps in Authentication:

**Step 1**: The user submits a request for authentication: By providing the cloud server with their identification, the user starts a session request, i.e., M_1_ = ID_i_. Authentication fails instantly if the identity is not present in the server’s database.

**Step 2:** A session key is generated and sent by the server: The cloud server creates a random session key (μkey) and obtains the matching ki after receiving IDi. The authentication parameter (yi) is then calculated as follows: yi = H(ki||μkey||ir), where H(·) is a secure hash function and ir is a randomly selected nonce to thwart replay attacks. The server then uses LigwEnc, a lightweight encryption technique, to encrypt the session key: LigwEnc(ki, μkey) = Yi.

The user is subsequently given the encrypted session key (Y_i_). If the encryption fails, the process is terminated.

**Step 3:** The user calculates the session key and responds: The user uses their saved key (k_i_) to decrypt (Y_i_), yielding μ_key via LigwDec(k_i_, Y_i_) = μ_key. A pre-defined validation function (f(·)) is used, together with y_i_, to compute another parameter (i_p) after the user chooses a random number (i_z). The user then uses μ_key to encrypt the combination of (i_p || i_z) and transmits Z_i_ = LigwEnc(μ_key, (i_p ||i_z)). Authentication is unsuccessful if decryption is unsuccessful or if i_p is inaccurate.

**Step 4**: Authentication is confirmed and verified by the server: To obtain i_z, the cloud server decrypts Z_i_ via LigwDec(μ_key, Z_i_) = (i_p|| i_z). It then recalculates LigwEnc(k_i_, μ_key, i_z) and returns the outcome to the user for final confirmation. The procedure fails and authentication is rejected if any of the decrypted values do not match the anticipated outcomes.

Implications for Security and Failure Management

- Authentication is refused immediately if ID_i_ is not present or does not match the stored credentials.
- The user must resubmit the request if Y_i_ decryption is unsuccessful because authentication cannot continue.
- Authentication is rejected if the user’s calculated IP address does not match the server’s expected value.
- A re-authentication procedure is initiated in the event of tampering or replay attacks.

**Figure 1:**
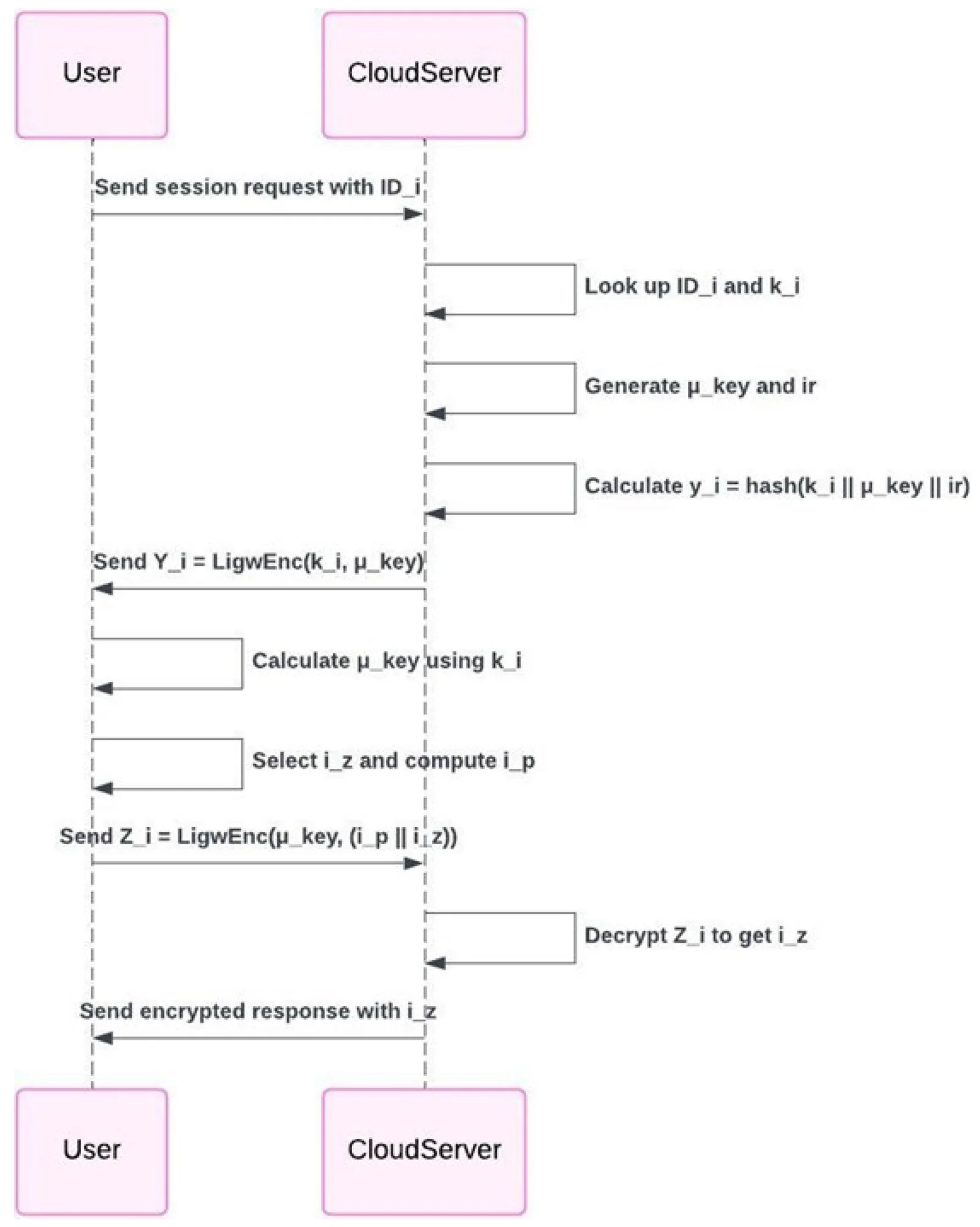
Authentication scheme model.

Figure 1 summarizes this four-step mutual-authentication workflow, from initial identification through server-side verification.

### 2.3. Layer-Wise and Systemic Authentication Security Challenges

In order to guarantee authorized access to IoT resources and keep unauthorized parties from jeopardizing system integrity, authentication is the first line of protection [19,20,21]. IoT authentication systems’ main goals are to:

- Verify user and device identities to stop impersonation attacks;
- Prevent replay attacks by guaranteeing data freshness and integrity;
- Preserve safe authentication in settings with limited resources.

However, IoT authentication techniques suffer from a number of security vulnerabilities because of memory restrictions, computational limitations, and difficulties with security updates. Three main layers can be used to methodically classify these risks:

#### 2.3.1. Physical Layer Authentication Challenges

IoT devices are susceptible to physical security concerns that have a direct influence on authentication processes, since they frequently operate in uncontrolled and hostile environments:

- Device Tampering and Cloning: Attackers can physically access IoT devices, extract cryptographic credentials, and clone them to circumvent authentication.
- Side-Channel Attacks (SCAs): By monitoring power usage, electromagnetic radiation, or timing variations during cryptographic processes, authentication keys can be inferred.
- Physical Destruction of Trusted Components: Authentication failures may result from the disablement or physical destruction of hardware authentication mechanisms such as Secure Elements (SEs) and Trusted Platform Modules (TPMs).

Implications for Authentication: Tampered devices posing as authentic nodes may cause data tampering; replay attacks using extracted credentials may jeopardize authentication integrity. 

Mitigation techniques: tamper-proof cryptographic modules and secure hardware components (such as PUFs, TEEs, and hardware tokens) can improve authentication security.

#### 2.3.2. Network Layer Authentication Challenges

Since the communication network is responsible for transmitting session keys and authentication information between devices, it is a popular target for network-based attacks:

- Eavesdropping and Credential Theft: Attackers obtain credentials without authorization by intercepting unencrypted authentication exchanges.
- Man-in-the-Middle (MITM) Attacks: Hackers impersonate legitimate users by altering authentication messages.
- Replay Attacks: Unauthorized access can be obtained by replaying captured authentication messages.
- Denial-of-Service (DoS) Attacks: These attacks stop legitimate devices from connecting by bombarding authentication servers with forged authentication requests.

Implications for Authentication: Authentication mechanisms with weak encryption enable unauthorized access; MITM attacks let attackers alter or forge authentication requests, compromising authentication integrity. Mitigation techniques include session-based authentication tokens, mutual authentication mechanisms (such as ECC, lightweight post-quantum cryptography, and blockchain-based authentication), and end-to-end encryption of authentication protocols.

#### 2.3.3. Application Layer Authentication Challenges

Access control and user authentication are handled by the application layer. Data sensitivity and diverse authentication schemes present a number of difficulties at this layer.

- Weak Credential Attacks and Password Policies: Because IoT authentication systems frequently use default credentials, they are susceptible to brute-force attacks.
- Credential Theft via Software and Ransomware: Attackers can install software that harvests authentication keys, allowing illegal access.
- Absence of Multi-Factor Authentication (MFA): This raises the possibility of identity spoofing and illegal access because many IoT systems do not enable MFA.

Implications for Authentication: IoT devices are vulnerable to widespread credential theft due to default passwords and inadequate authentication mechanisms; authentication can be disabled via malware-based credential hijacking, which compromises the entire system. Mitigation techniques: blockchain-based identity management, biometric authentication, and AI-driven intrusion detection improve authentication security.

#### 2.3.4. General Challenges in IoT Authentication

IoT authentication confronts basic security issues in addition to layer-specific threats:

1. Limited capacity for computation and storage

The resources of IoT devices are insufficient to carry out computationally demanding authentication procedures.

Because they demand a lot of computing power, traditional authentication methods like RSA and Diffie–Hellman key exchange are inappropriate for the Internet of Things.

Mitigation techniques include lightweight authentication methods such as optimized hash-based authentication schemes, Elliptic Curve Cryptography (ECC), and Lightweight Post-Quantum Cryptography (L-PQC) [22,23,24].

2. Energy Efficiency and Resource Limitations: IoT devices frequently switch to power-saving modes and have short battery lives, which can interfere with authentication [19,23,24].

Energy-efficient cryptographic approaches such as PUF-based authentication, lightweight hash algorithms (SHA-256 and Blake2), and XOR operations are examples of mitigation strategies.

Scalability and dynamic security updates: To fix vulnerabilities and integrate new devices, IoT systems regularly upgrade authentication frameworks [22,23,24]. One of the biggest challenges is handling dynamic authentication updates without sacrificing security.

Mitigation techniques include secure over-the-air (OTA) authentication updates and decentralized identity management using blockchain-based authentication.

## 3. IoT Authentication Technologies

### 3.1. Introduction to Authentication Technologies

**Figure 2:**
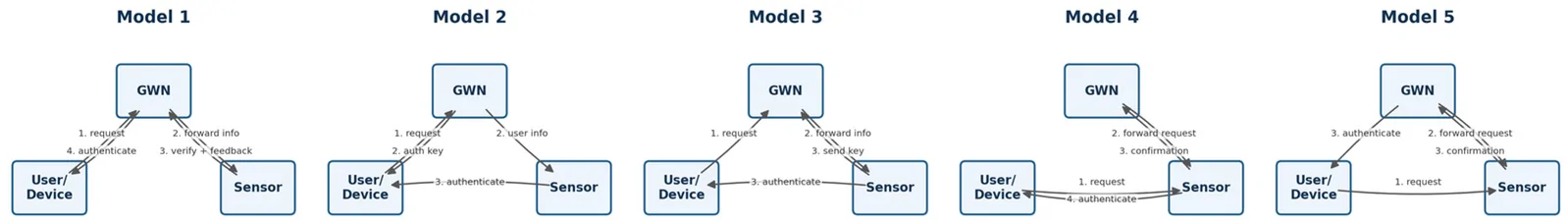
IoT network authentication model. The five sub-panels separate the message flow of each authentication model listed in Table 1 (User/Device → GWN → Sensor).

In IoT applications, identity authentication is used to identify users and devices and restrict unauthorized users from manipulating devices. In the IoT, components can communicate with each other and share data, which requires each user or device to authenticate other objects and devices. Several commonly used identity authentication models are presented in the [25], as shown in Figure 2 and described in Table 1. In most cases, gateway nodes (GWNs) cannot directly authenticate users using the information they send. Remote sensor nodes assist in the authentication process when direct authentication is not feasible. Moreover, cross-domain device authentication in IoT presents a significant challenge. Existing solutions relying on usernames and passwords or a single authentication method may not be suitable due to IoT’s heterogeneity, as they remain vulnerable to attacks [26]. IoT devices have constraints such as low power consumption, limited memory, mobility, and multi-network protocol support [27]. Hence, authentication technology is a critical component of IoT security, ensuring that only trusted devices participate in network operations [28]. Earlier survey work has similarly catalogued the range of security weaknesses inherent to IoT authentication mechanisms [22].

### 3.2. Types of IoT Authentication Protocols

In the Internet of Things (IoT), identity authentication is critical for ensuring secure communication and preventing unauthorized access to devices and networks. Unlike traditional authentication mechanisms designed for general computing environments, IoT authentication protocols must address specific constraints, including low power availability, limited computational resources, and heterogeneous device architectures. Given these challenges, researchers have developed various identity authentication mechanisms tailored to IoT environments. Based on [29], these authentication protocols can be categorized into password-based authentication, MAC address-based authentication, public identity-based authentication, token-based authentication, and biometric authentication [30]. Each of these protocols has distinct foundational principles, advantages, and limitations, as discussed in detail below.

#### 3.2.1. Password-Based Authentication

Foundational Aspects: Password-based authentication is one of the oldest and most widely used authentication techniques. It requires users or devices to provide an ID–password pair, which is compared against stored credentials in an authentication server. This authentication method relies on cryptographic hashing functions to securely store and verify passwords, thereby preventing unauthorized access. Additionally, secure password management mechanisms, such as salted hashing and multi-factor authentication (MFA), enhance resilience against common attacks.

Limitations: Despite its widespread use, password-based authentication has several drawbacks:
Security Vulnerabilities: Password-based authentication is susceptible to brute-force attacks, phishing, and password leaks.
Usability Issues: It requires users to remember complex passwords, often leading to weak passwords or reuse across multiple platforms.
Scalability Constraints: As the number of users grows, maintaining secure password databases increases computational and storage overhead.
Unsuitability for IoT: Many IoT devices lack user interfaces, making password entry impractical.

Table 2 summarizes the computational overhead, energy efficiency, scalability, and security resilience of password-based authentication.

#### 3.2.2. MAC Address-Based Authentication

Foundational Aspects: MAC address-based authentication relies on the unique Media Access Control (MAC) address assigned to each network interface. The authentication server maintains a database of approved MAC addresses and grants access to devices whose MAC addresses match stored records. This method is simple, lightweight, and effective for device authentication in local network environments [31].

Limitations:

- Security Risks: Easily susceptible to MAC address spoofing, replay attacks, and unauthorized cloning.
- Lack of Encryption: Unlike cryptographic authentication methods, MAC address verification does not inherently offer data encryption or integrity validation.
- Limited Scalability: The increasing number of IoT devices may exceed the MAC address pool, requiring a new address format standard.
- Not Suitable for Public Networks: This method works well in closed networks but lacks security mechanisms for large-scale IoT deployments.

Table 3 summarizes the computational overhead, energy efficiency, scalability, and security resilience of MAC address-based authentication.

#### 3.2.3. Public Identity-Based Authentication

Foundational Aspects: Public identity-based authentication leverages asymmetric cryptography, where public and private key pairs are used to authenticate users and devices. A public key is associated with an identity attribute (e.g., email or IP address), and a corresponding private key ensures secure authentication. This scheme eliminates the need for pre-shared secrets, making it more scalable for distributed IoT environments. Elliptic curve cryptography (ECC) has been widely adopted to implement this class of scheme efficiently for IoT and cloud-server settings, keeping key sizes and computational overhead manageable relative to other public-key approaches [21].

Limitations:

- Computational Complexity: Public-key cryptographic operations, such as RSA and ECC, require significant processing power, making them impractical for resource-constrained IoT devices.
- Energy Consumption: Cryptographic computations are power-intensive, reducing battery life in IoT deployments.
- Key Management Challenges: Secure distribution and storage of private keys are critical but challenging in dynamic IoT environments.
- Susceptibility to Identity Spoofing: If the key infrastructure is compromised, attackers can assume false identities.

Table 4 summarizes the computational overhead, energy efficiency, scalability, and security resilience of public identity-based authentication.

#### 3.2.4. Token-Based Authentication

Foundational Aspects: Token-based authentication uses dynamically generated authentication tokens, such as one-time passwords (OTPs) or cryptographic keys, to verify users and devices. This approach ensures enhanced security and prevents replay attacks. Token-based methods include one-time passwords (OTPs) sent to a registered device for authentication and hardware tokens such as USB dongles, smart cards, or RFID tags that store authentication credentials.

Limitations

- Storage and Validation Overhead: Tokens must be securely stored and validated, increasing processing requirements.
- Dependency on Communication Channels: OTP mechanisms require reliable communication networks for secure transmission.
- Device Loss or Theft: Hardware tokens can be lost or stolen, leading to unauthorized access risks.

Table 5 summarizes the computational overhead, energy efficiency, scalability, and security resilience of token-based authentication.

#### 3.2.5. Biometric Authentication

Biometric authentication verifies users based on unique physiological or behavioral traits, such as fingerprints or facial features. It is a security mechanism that leverages distinctive characteristics of individuals for identity verification. This authentication method employs specialized biometric sensors to capture, process, and compare biological data against stored reference templates. Due to its inherent uniqueness, biometric authentication offers improved security and resistance against conventional attack vectors such as eavesdropping and impersonation [32,33]. However, challenges related to spoofing, hardware dependency, and operational constraints necessitate further advancements in this domain. Table 6 provides a comparative evaluation of different biometric authentication techniques based on computational overhead, energy efficiency, scalability, and security resilience.

- Biometric Authentication Techniques: Several biometric modalities are utilized in authentication frameworks, each offering distinct advantages and limitations:
- Fingerprint Recognition: As one of the most widely implemented biometric techniques in IoT-based security systems, fingerprint authentication involves capturing and analyzing ridge and valley patterns for identity verification. Recent advancements in multimodal biometric systems have integrated fingerprint data with additional biometric traits to enhance robustness and security [34].
- Facial Recognition: This technology extracts and analyzes facial feature vectors to authenticate users. While extensively used in mobile and surveillance systems, it remains susceptible to spoofing via deepfake attacks. Hybrid deep-learning frameworks have been introduced to enhance recognition accuracy under varying lighting and environmental conditions [35].
- Iris and Retina Recognition: Iris recognition employs mathematical feature extraction techniques to analyze the unique structural patterns of the iris, whereas retina recognition maps blood vessel formations for authentication. These methods offer high accuracy and resistance to forgery but require specialized imaging hardware, leading to increased implementation costs [36].
- Electroencephalogram (EEG)-Based Authentication: EEG-based authentication utilizes brainwave activity as a biometric identifier, offering a highly secure mechanism resistant to spoofing. Deep learning models have demonstrated improved classification of EEG signals for continuous authentication [37].
- Photoplethysmography (PPG)-Based Authentication: PPG-based authentication relies on blood volume pulse signals to verify identity. Due to its non-invasive nature, this technique has been increasingly integrated into wearable IoT security systems for real-time user authentication [38].
- Gait Recognition: Gait authentication utilizes motion sensors and machine learning algorithms to analyze walking patterns. This method is promising for continuous authentication in wearable and mobile security systems but can be affected by changes in walking behavior due to injuries or environmental conditions [39].
- Breathing Pattern Recognition: This authentication method relies on respiratory signals, captured using microphones or radar sensors, to differentiate individuals. It has been studied for its applicability in passive and continuous authentication for IoT-based healthcare applications [40].
- Voice-Based Authentication: Voice biometrics analyze speech patterns for authentication. While convenient and widely used, it is susceptible to spoofing attacks such as voice cloning or playback attacks. Advanced AI-based voice anti-spoofing techniques are being explored to counter these vulnerabilities [41].
- Multimodal Biometric Authentication: This method integrates multiple biometric traits (e.g., fingerprint and voice) to improve system accuracy, resilience, and adaptability. Studies have explored the fusion of deep learning-driven feature extraction techniques to enhance multimodal biometric security frameworks [42].

##### Security Considerations and Challenges

- Hardware Dependency: The deployment of biometric systems necessitates specialized sensors, leading to increased infrastructure costs.
- Operational Constraints: Recognition performance is affected by environmental conditions such as lighting variations in facial recognition systems and background noise in voice-based authentication.
- Spoofing and Privacy Risks: Advances in AI-driven forgery techniques, including deepfake-based attacks, raise concerns regarding the security of facial and voice biometrics. Novel countermeasures such as liveness detection and adversarial machine learning techniques are being explored [43].

Ongoing research efforts aim to develop highly secure, resilient, and hard-to-spoof biometric authentication mechanisms by integrating advanced AI models and cryptographic techniques within IoT security frameworks. Table 7 presents a comparative summary of the five authentication classes covered in this survey, highlighting computational overhead, energy efficiency, scalability, security resilience, and suitability for IoT applications.

Token-based authentication provides the best trade-off among the schemes surveyed for security, scalability, and energy efficiency, making it the most broadly deployable choice for general-purpose IoT. Concretely, token-based schemes are the most flexible because they require only lightweight symmetric-key operations and short-lived/non-reusable credentials; by avoiding the computationally expensive public-key computation required by public-identity schemes and/or the specialized sensor hardware required by biometric schemes, token credentials still provide resistance to replay attacks by virtue of being refreshed frequently (Table 5). Token-based auth thus provides the best overall baseline/default for constrained endpoints, given current technology, i.e., it is not objectively the most secure scheme possible. Table 6 and Table 8 demonstrate that various biometric modalities and public-identity cryptography can offer higher raw resilience/security entropy against attacks. However, the computational/financial cost of supporting these classes makes them untenable for most resource-constrained IoT devices. Said another way, there is no silver bullet. However, this status quo where token-based credentials lead only in the specific context of constrained endpoints, while schemes from the stronger classes are prohibitively expensive in that context, should, itself, serve as motivation for why the research field needs hybrid schemes (that combine token factor types with biometric and cryptographic factors; see Section 4.5), lightweight primitives that reduce the costs associated with the latter two classes, and quantum-safe (post-quantum) mechanisms that maintain those long-term security guarantees when large-scale quantum attacks become feasible. In contrast, MAC-based authentication is highly vulnerable to spoofing and lacks intrinsic security. Public identity-based authentication offers good scalability but demands high computational power, making it impractical for the most constrained endpoints. While biometric authentication ensures high security, it requires significant computational resources and energy-efficient implementations for IoT feasibility. The introduction of implicit authentication techniques, such as gait and breathing-pattern recognition, offers promising alternatives for continuous authentication with lower user effort. Section 3.2.6, Section 3.2.7, Section 3.2.8 and Section 3.2.9 below express this comparison in quantitative, information-theoretic and confusion-matrix terms rather than qualitative labels alone, and Section 5 revisits the token-based claim in light of that quantitative evidence.

#### 3.2.6. Quantitative and Information-Theoretic Comparison of Authentication Mechanisms

The comparisons in Table 2, Table 3, Table 4, Table 5, Table 6 and Table 7 are qualitative because password-, MAC-, token-, and biometric-based schemes in the underlying literature are evaluated using nonuniform experimental setups, hardware, and threat models. Accordingly, a single head-to-head numerical benchmark across all four IoT domains is impossible in the scope of this comparative survey. Responding to reader requests for a more mathematical and scientific treatment, this subsection formalizes three quantitative lenses through which recent quantitative IoT- security surveys compare mechanisms: information-theoretic entropy, confusion-matrix-based classification metrics, and receiver operating characteristic (ROC) reasoning. We reinterpret Table 2, Table 3, Table 4, Table 5, Table 6 and Table 7 using these lenses rather than present new unpublished experimental measurements.

#### 3.2.7. Entropy as a Measure of Credential Unpredictability

For a credential space with n possible values occurring with probabilities of p_1_,…, p_n, the Shannon entropy (H = −Σ p_i log_2_ p_i, in bits) measures the unpredictability of a credential in response to an attacker mounting a brute-force/dictionary-style guessing attack; an n-bit key with uniformly distributed values has a maximum entropy of n bits, while a human-selected password chosen from a small biased dictionary has much lower effective entropy. Recent systematic surveys of lightweight cryptography for IoT make explicit use of entropy, along with execution time and energy consumption, as axes for quantitative comparison among cryptographic and authentication schemes [12]. Table 8 translates the qualitative entries in the “security resilience” column of Table 7 to the approximate credential-entropy classes found in that literature.

#### 3.2.8. Confusion-Matrix Metrics for Biometric and Behavioral Authentication

Biometric and implicit behavioral schemes are conventionally evaluated with a 2 × 2 confusion matrix computed over genuine versus impostor authentication attempts. The familiar false acceptance rate (FAR = FP/(FP + TN)), false rejection rate (FRR = FN/(FN + TP)), and true positive (genuine acceptance) rate (TPR = TP/(TP + FN)) are derived from the confusion matrix. Sweeping the decision threshold and plotting TPR vs. FAR produces the receiver operating characteristic (ROC) curve; the point on the ROC curve where FAR = FRR is called the equal error rate (EER). This single-number summary is perhaps the most commonly used metric in the biometrics literature for comparing modalities and thus represents a natural companion to the qualitative “security resilience” ratings presented above in Table 6.

Arranged according to the qualitative ratings of Table 6, the modalities considered here rank from lowest to highest expected EER (i.e., weakest to strongest discrimination), approximately as follows: voice ≈ gait ≈ facial recognition (high EER) < fingerprint ≈ PPG (medium EER) < iris/retina < multimodal fusion ≈ EEG (low EER). While this ranking obviously agrees with the security-resilience column from Table 6 repeated here for convenience, it is meant to serve as something of a translation to the quantitative metrics sought during review. Should this chart be used for rough engineering estimates, it would be wise to consult modality- and dataset-specific EER numbers first, as EER depends sensitively on sensor quality, population, and spoofing countermeasures [12,13].

#### 3.2.9. A Combined Overhead-Versus-Resilience View

**Figure 3:**
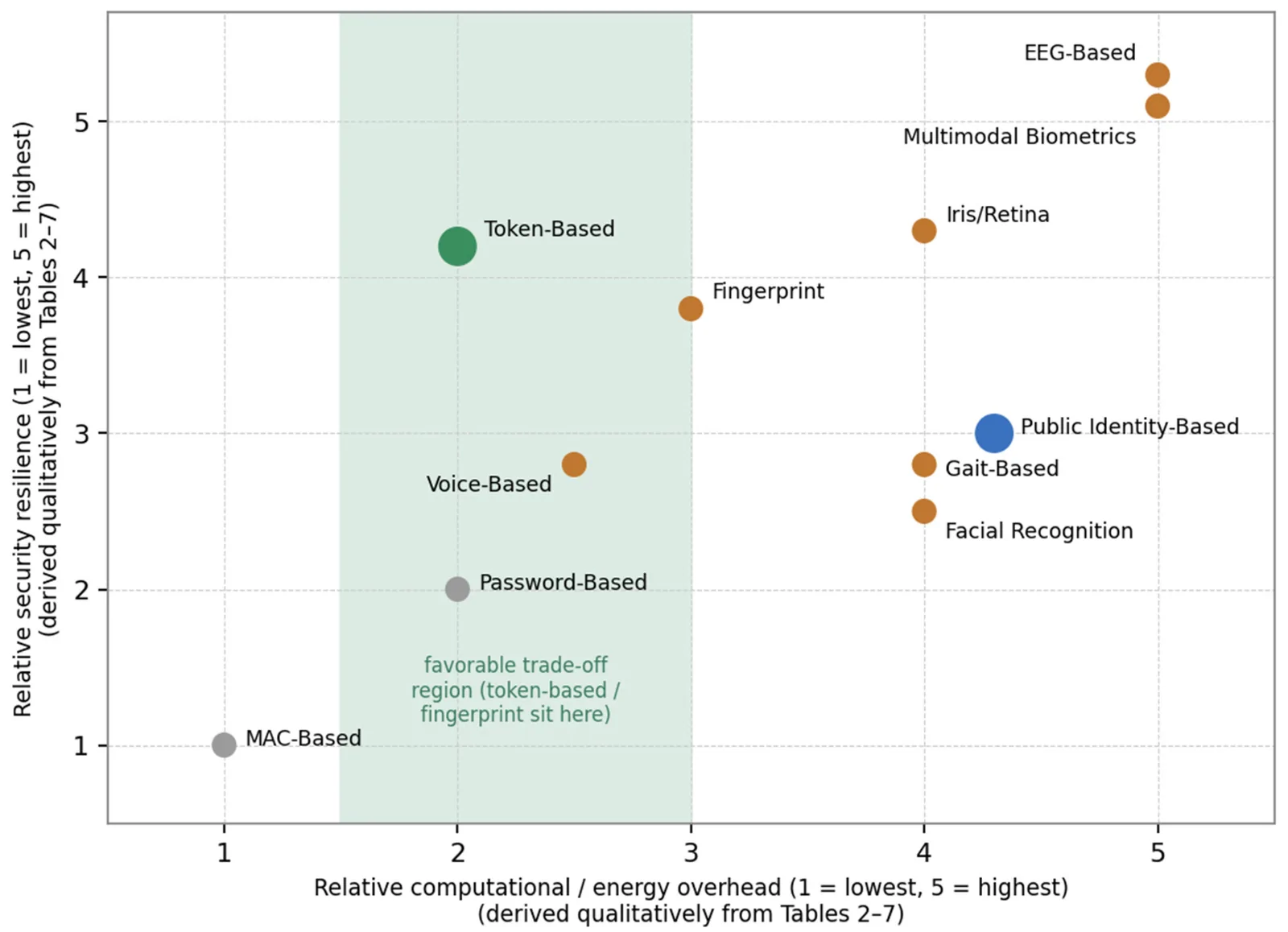
Conceptual overhead-versus-resilience positioning of IoT authentication classes, synthesized from Table 2, Table 3, Table 4, Table 5, Table 6 and Table 7 and the entropy/EER reasoning in Section 3.2.7 and Section 3.2.8.

Figure 3 situates the five authentication classes and the biometric sub-modalities along two axes: relative computational/energy overhead (computed from Table 2, Table 3, Table 4, Table 5, Table 6 and Table 7) and relative security resilience (defined above in terms of entropy and EER). This plot is a qualitative overlay meant to expose the trade-off structure; it does not represent any newly measured benchmark data, and the relative positions of each category should be interpreted as ordinal, not cardinal. Both token-based authentication and fingerprint recognition lie in the ‘sweet spot’ of moderate overhead and above-average resilience, reinforcing the qualitative conclusion reached at the end of Section 3.2. Multimodal biometrics and EEG-based authentication are the most resilient but come at the highest overhead. Finally, MAC-based authentication has the lowest overhead but also provides the lowest security, recreating the trade-off pattern seen in Table 7 here in one visualization.

#### 3.2.10. Higher-Layer Authentication Architectures: Federated Identity, Single Sign-On, and OAuth

Section 3.2 and Section 3.3 cover authentication mechanisms at the device and protocol levels, respectively. An orthogonal architecture that addresses how identities are issued, delegated, and trusted across organizational and domain boundaries was identified as a significant omission in previous surveys of IoT authentication. Enterprise identity interoperability has become an important requirement, as most IoT deployments will eventually reside behind a Web, mobile, or cloud front end. This subsection provides an overview of three such architectures and explains their relevance to the RFID, smart grid, IoV, and smart-home use cases covered in Section 3.3.

#### 3.2.11. Federated Identity Management (FIM)

In federated identity management (FIM), once authenticated by a trusted identity provider (IdP), a user or device can reuse that authentication assertion across many independently administered service providers rather than maintaining separate credential stores for each service offering. Fremantle and Aziz [44] were among the first to tailor FIM to IoT settings, proposing a federated identity and access-management architecture for constrained devices specifically designed to have low runtime overheads and later expanding on it with a privacy-preserving federation scheme (“OAuthing”) that minimizes downstream-service exposure of identity information [45]. More recent self-sovereign identity (SSI) proposals take this concept even further, allowing a device/user to instead store verifiable credentials in a personal wallet rather than relying on a centralized IdP [46]. This alleviates the single-point-of-failure concern of traditional FIM and nicely complements the blockchain-based IDM approaches discussed earlier in Section 4.3.

#### 3.2.12. Single Sign-On (SSO)

SSO (“Single Sign-On”) is likely the most prevalent user-facing feature implemented as a result of an FIM deployment: once you have proven your identity to one entity in the network, you should be able to access other related services without re-authenticating yourself to each one independently. In the context of IoT, SSO tends to be applied highest up in the stack, at the gateway and application-service layers (Section 2.1), rather than at the constrained end device itself. This is because cryptographic handshakes required for SAML- or OIDC-based SSO are generally too heavyweight for the nodes discussed in Section 2.3.4 and Section 3.2. As a consequence, SSO is likely to find its place highest in the IoT stack as well: as an authentication convenience layer for human operators and dashboards that manage smart grids, IoV fleets, or smart-home gateways rather than replacing the device-level mechanisms summarized in Table 7.

#### 3.2.13. OAuth 2.0/OpenID Connect for IoT

OAuth 2.0 is an authorization protocol that allows a client or device to acquire a scoped, time-limited access token without ever possessing the resource owner’s long-term credentials. OpenID Connect builds on OAuth to authenticate the end-user, in addition to authorizing an application. IoT-OAS [47] is a proposal for an OAuth-based authorization-service architecture that offloads authorization decisions from constrained IoT devices to a dedicated authorization server, and Fremantle et al. [44] evaluated the use of OAuth 2.0 in combination with lightweight MQTT as a means for enabling token-based delegation to machine-to-machine (M2M) IoT devices. The results of these studies suggest that OAuth/OIDC is only appropriate for gateway-, cloud-, and application-layer authorization in smart-home and smart-grid scenarios from Section 3.3 where a head-end system can handle the token issuance and validation workload on behalf of a fleet of constrained devices behind it. Known attack surfaces specific to OAuth such as access-token theft, cross-site request forgery, and “path confusion” between authorization servers [48] are exacerbated when used with IoT devices and must still be mitigated using short-lived tokens, PKCE, and strict validation of redirect URIs as recommended by OAuth 2.1 [48].

Overall, FIM, SSO, and OAuth/OIDC do not overlap with any of the five device-level classes considered in Table 7; instead, they reside one layer up, and mature IoT deployments often involve a combination of a lightweight device-level mechanism (e. g., token- or PUF-based authentication happening at the sensor node itself) with an OAuth/OIDC-based federation layer located at gateway or cloud boundary. We view the two-layer combo as one of hybrid authentication flavors discussed below in Section 4.5.

### 3.3. Comparison of Different Application Authentication Technologies

#### 3.3.1. RFID Technology

An RFID tag and an RFID reader are the standard components of RFID, a wireless technology. RFID is extensively utilized in industries like climate monitoring, healthcare, and supply chain management [28]. Complex encryption techniques cannot be used for identity identification because of the RFID-enabled IoT devices’ poor processing capability, short storage capacity, and simplistic CPU structure; for this reason, reciprocal (mutual) identity-authentication approaches specifically designed for RFID-based IoT applications have been proposed in the literature [41,42]. Lightweight encryption algorithms are used to improve security storage capacities, speed up authentication, and lower computing costs. A lightweight RFID identity authentication system for anonymity in the Internet of Things was proposed by Gope et al. [23]. Four components make up the network model on which the system is based: an RFID tag, a reader, and two servers (the backend database and authorized cloud). This scheme employs unlinkable anonymous identities, backup keys, and hash functions to thwart replay, forgery, cloning, denial-of-service (DoS), and location-tracking attacks. It also offers scalability, tag anonymity, mutual authentication, and defense against bogus data-injection attacks. One efficient way to ensure privacy is through mutual authentication. When RFID tags and readers use mutual authentication, device tag data is processed using anonymization techniques, and effective authentication is made possible using lightweight encryption algorithms. Both anonymization and lightweight encryption can be accomplished in future studies using algorithms such as elliptic curve encryption. Related work has also proposed lightweight, privacy-preserving RFID authentication designed for distributed IoT infrastructure with secure localization services in smart-city deployments [20].

#### 3.3.2. Smart Grids

Although smart grids are more convenient and efficient than traditional electricity grids, they nevertheless have a number of security issues. Lightweight cryptographic authentication approaches based on Merkle trees and HMAC (Hash-based Message Authentication Code) are presented in the literature [40,41]. Lightweight Diffie–Hellman cryptosystems are used [37] to accomplish mutual authentication between smart meters and other system elements. Numerous academics are investigating low-storage-cost cryptographic algorithms and lightweight key management to address performance and security issues. The use of HMAC and homomorphic encryption for multicast authentication in smart grids is covered in the literature [49,50]. This technique minimizes data sharing and protects privacy by hiding the identity of smart meters. It is evident from the aforementioned studies that establishing lightweight authentication in smart grids requires the use of lightweight encryption methods. The following cryptographic techniques can be used in smart grid systems’ lightweight authentication strategies based on privacy considerations: (1) basic bit operations; (2) a mix of hash functions with random integers or timestamps; and (3) lightweight encryption algorithms, including elliptic curve encryption.

#### 3.3.3. Internet of Vehicles (IoV)

The Internet of Vehicles (IoV) is created by connecting automobiles to the Internet. In IoV systems, vehicle authentication is a difficult problem. A collaborative message-authentication technique that minimizes authentication latency and lowers message-authentication overhead is proposed in [51]. An authentication technique that allows requests from several vehicles to be verified in batches rather than one at a time is presented in [52]. Ref. [41] presents a batch-authentication method based on a modified DSA (Digital Signature Algorithm) and ECDSA (Elliptic Curve Digital Signature Algorithm) that makes the authentication process up to seven times faster than individual authentication; this method uses multiple signatures generated by different signers or a single signer. One of the main concerns with IoV is ensuring the privacy and security of vehicle identity data, which calls for the creation of cryptographic systems that can protect privacy and ensure mutual authentication.

#### 3.3.4. Smart Homes

In order to authenticate and communicate with smart-home equipment, users use mobile devices or personal computers. This allows for remote control, monitoring, and access. By using the shared physical location of devices as a shared secret, Miettinen et al. [53] present a context-based device mutual-authentication protocol that does not require password input and has a number of usability advantages over competing systems. To guarantee data security, Sun et al. [54] created a method that enables smart-home users to connect securely to terminal devices remotely via a network; this connection is also made possible by mutual authentication between communication parties. Based on the lightweight characteristics of the Constrained Application Protocol (CoAP), an application-layer protocol for client–server communication, Jan et al. [55] presented an IoT mutual-authentication mechanism. Through the exchange of encrypted messages with a 256-bit payload, the client and server may verify each other’s identities thanks to this protocol, which leverages the benefits of AES encryption to provide a secure communication channel. More recent work in smart homes continues this trend: Kokila and Reddy [36] surveyed authentication, access control, and scalability models for IoT security, and Rajeh et al. [56] very recently proposed a lightweight, biometric-driven smart-home authentication scheme that encodes a one-time Iris code and fuses it with cryptographic hashing to attain mutual authentication between the user, gateway, and device in under 2 milliseconds of computation time and ~2400 bits of communication overhead, formally verified using the Real-Or-Random model—a concrete, dated example of the biometric/lightweight-cryptography hybrid approach recommended by this survey in Section 4.5. A further 2026 proposal continues in this direction by outlining a scalable smart-home IoT architecture that integrates a Web-based remote control system with biometric access control, thereby presenting the same Web front end + biometric pattern for gateway- and application-layer authorization as mentioned in Section 3.2.13 [57]. IoT identity-authentication solutions across several application domains are compiled in Table 9, which also highlights their authentication protocols and classifies them using the classification methodology described in this section. According to the research, new cryptographic technologies or algorithms are not needed to achieve mutual authentication in these protocols; nevertheless, creating an authentication system that works with resource-limited IoT devices remains a novel and open task.

## 4. Emerging Trends in IoT Security Authentication Mechanisms

**Figure 4:**
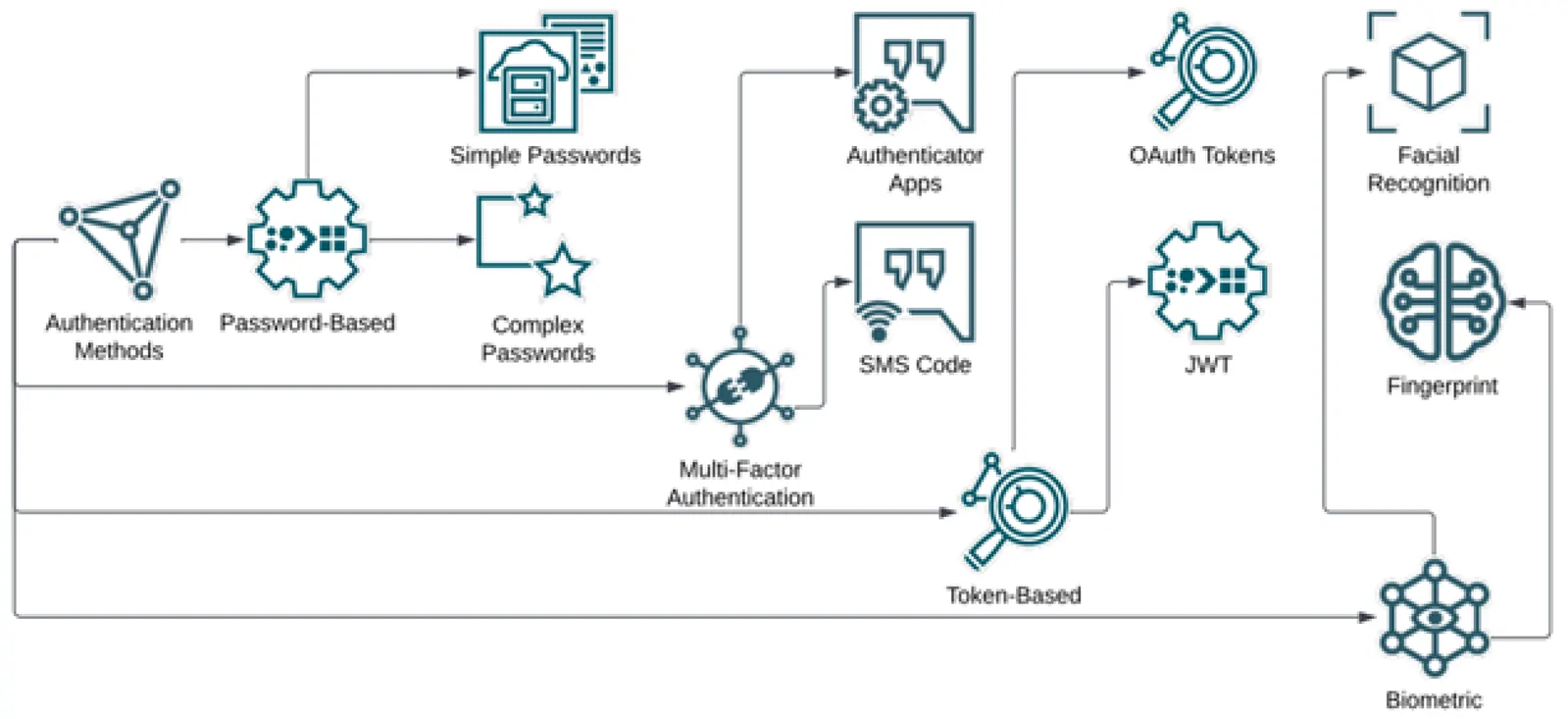
Classification of identity authentication in the Internet of Things (IoT).

Research on reliable and scalable authentication methods is still crucial as the Internet of Things (IoT) ecosystem grows. Because of the resource restrictions of IoT devices, traditional authentication methods—such as password-based and cryptographic key-based approaches—frequently show shortcomings. Recent developments in authentication techniques have concentrated on trusted computing frameworks, blockchain-based identity management, and implicit biometric authentication in order to overcome these difficulties. The methods, benefits, and difficulties of these new trends are covered in this section. The classification of identity-authentication techniques discussed throughout this survey—spanning password-based, multi-factor, token-based, and biometric mechanisms—is summarized in Figure 4.

### 4.1. Implicit Biometric Authentication in IoT Security

In IoT environments, implicit biometric authentication has drawn interest as a potential ongoing, non-intrusive identity-verification method. Implicit biometric authentication uses passive physiological and behavioral characteristics to verify identity, in contrast to explicit biometric authentication techniques (such as fingerprint or facial recognition), which necessitate direct user input [32,33]. This method reduces authentication friction and user effort while improving security.

#### 4.1.1. Key Implicit Biometric Authentication Techniques

**Keystroke Dynamics:** Keystroke dynamics examine a user’s distinct typing habits, including typing speed, inter-key delay, and key-press time. In IoT-based applications and smart devices, these behavioral characteristics can be used for passive authentication [34].
**Gait Recognition:** To identify people based on their walking patterns, gait-based authentication uses motion sensors (such as accelerometers and gyroscopes) built into wearable technology. Deep learning-based gait detection for continuous authentication has been shown to be feasible in recent research [35].
**Electroencephalogram (EEG)-Based Authentication**: EEG-based authentication offers extremely secure, person-specific authentication by using brainwave patterns recorded by wearable headsets. This approach is particularly useful in medical IoT (MIoT) applications [36].
**Photoplethysmography (PPG)-Based Authentication**: PPG-based authentication measures blood-volume pulse signals using optical sensors found in fitness trackers and smartwatches. This provides a non-intrusive, continuous authentication mechanism, leveraging cardiovascular patterns [37].

#### 4.1.2. Challenges in Implicit Biometric Authentication

Implicit biometrics have many benefits; however, there are a few issues that need to be resolved:

- Sensor Reliability and Environmental Variability: The precision of biometric data can be impacted by elements including ambient light, motion noise, and device positioning.
- Privacy and Data Security Issues: Constant biometric data monitoring creates privacy issues that call for the application of privacy-preserving strategies like differential privacy and federated learning.
- Computational Overhead and Real-Time Processing: Because IoT devices frequently have little processing power, lightweight AI models for biometric identification must be created.
- Spoofing Attack Vulnerability: To identify and stop spoofing and replay attacks against biometric authentication systems, sophisticated adversarial machine learning algorithms are needed.

### 4.2. Industry-Led Authentication Solutions

A number of IT companies are creating authentication frameworks specifically for the security of the Internet of Things [60,62,63,64]:

- For device-level mutual authentication, Alibaba Cloud’s Link ID2 employs a hybrid authentication approach that blends certificate-based asymmetric cryptographic schemes with pre-shared symmetric key techniques.
- Tencent’s User Security Infrastructure (TUSI) proposes a Public Key Infrastructure (PKI)-based authentication standard for IoT applications, employing asymmetric cryptography for identity management.
- In extensive IoT deployments, these frameworks seek to improve interoperability, robustness, and authentication security.

### 4.3. Blockchain-Based Authentication for IoT Security

For IoT networks, blockchain technology has become a decentralized, tamper-resistant authentication system [38]. The following are the main characteristics of blockchain that support IoT security [65,66,67]:

- Immutability maintains the integrity of identity data by preventing unwanted changes to authentication records; decentralization removes single points of failure that are inherent to centralized authentication servers.
- Cryptographic Security uses elliptic curve cryptography (ECC) and hash functions (SHA-256, RIPEMD-160, etc.) to manage keys securely. Devices may independently verify identities by incorporating blockchain into IoT authentication protocols, reducing the possibility of identity spoofing, man-in-the-middle attacks, and unwanted access.

Blockchain-backed designs of this kind have been applied to secure industrial-IoT communication [70] and to build privacy-preserving authentication protocols for general IoT applications [71], and blockchain-empowered IoT platforms have similarly been proposed to support automation across multiple industry sectors [72]. In parallel, deep learning has been combined with blockchain-style verification to strengthen IoT authentication against evolving attack patterns [73].

### 4.4. Trusted Computing and Next-Generation Authentication Models

Authentication methods are moving toward hardware-based security designs with the introduction of Trusted Computing 3.0. These consist of:

- Secure Boot Mechanisms: Make sure that IoT devices only run verified firmware.
- Remote Attestation Protocols: These allow for ongoing device integrity checks against pre-established security guidelines.
- 5G-Enabled Security Frameworks: Using edge computing and AI-driven anomaly detection, these frameworks improve device attestation and real-time authentication [39].

### 4.5. Future Research Directions in IoT Authentication

Authentication methods need to evolve alongside IoT security threats in order to offer effective, scalable, and durable identity-management solutions. Future studies ought to concentrate on:

- Hybrid Authentication Modeling: Creating multi-layered authentication systems that combine behavioral, biometric, and cryptographic authentication methods, potentially combined with the OAuth/OIDC federation layer discussed in Section 3.2.13;
- AI-Driven Authentication: Using anomaly detection based on machine learning to instantly identify fraudulent authentication attempts;
- Post-Quantum Cryptography (PQC): Researching encryption techniques that are resistant to quantum-computing attacks in order to guarantee long-term security in post-quantum computing settings.

Next-generation authentication models can greatly improve IoT security by combining trusted computing frameworks, blockchain-based identity management, and implicit biometric authentication [68,74,75,76,77]. These developments will reduce emerging cybersecurity threats in IoT deployments by enabling smooth, continuous, and tamper-resistant identity verification.

### 4.6. Summary of Recently Proposed Authentication Mechanisms (2022–2026)

To provide a direct answer to what new authentication mechanisms have been proposed recently and how they have been applied across the four IoT domains covered in this survey, Table 10 (new) lists representative schemes published after 2022 that were identified using the search strategy described in Section 1.3 but not already included in Table 2, Table 3, Table 4, Table 5, Table 6 and Table 7. They are presented using the same five-class taxonomy throughout the survey so that they may be read as additions to Table 2, Table 3, Table 4, Table 5, Table 6 and Table 7, updated with publication dates rather than as a separate list of literature.

Two patterns are visible from this latest crop of work. First, very few 2023–2026 proposals focus solely on proposing an entirely new cryptographic primitive; rather, most tack an existing lightweight primitive (hashing, XOR, or symmetric encryption) onto a second authentication factor—whether a wearable-fused biometric, rotating one-time biometric passcode, or even explicit quantum-computing threat model—which complements the hybrid approach already endorsed in Section 4.5. Second, even the reviews themselves have improved: recent IoT-authentication surveys [12,13,14] now include explicit search strings, inclusion/exclusion criteria, and quantitative comparison metrics—methodologies this revision follows in Section 1.3 and Section 3.2.6, Section 3.2.7, Section 3.2.8 and Section 3.2.9.

## 5. Conclusions

Based on the commonalities and essential features of IoT authentication protocols, this article provides an overview and analysis of the main IoT identity-authentication systems. Three characteristics that IoT security authentication protocols should include are mutual authentication, privacy protection, and a lightweight design [77]. Based on various application scenarios and IoT device security capabilities, the features of various IoT authentication protocols are examined in this work, and security requirements are suggested in response to a range of external threats. The goal of this classification is to establish a safe Internet of Things environment.

To restate the trade-off claim using the terminology from Section 3.2.6, Section 3.2.7, Section 3.2.8 and Section 3.2.9, token-based authentication is not claimed to provide the highest possible security mechanism in absolute terms—Table 6 and Table 8 demonstrate that multiple biometric modalities (fusion and EEG) and public-identity cryptography have higher nominal security resilience and entropy values. The claim is both narrower and relative: of the five surveyed classes, token-based schemes fall within the desirable overhead-versus-resilience region shown in Figure 3 (new)—low to moderate computational and energy overhead (Table 5), along with above-average resilience against security attacks (strong, replay-resistant security in the form of short-lived credentials, which are never reused) and high scalability (Table 7)—which is why it is suggested to be used as the most appropriate general-purpose default for constrained IoT endpoints, while biometric and public-identity options should be used where their additional overhead is justified (either at less-constrained points like gateways or in application domains like healthcare IoT and vehicular fleets where that resilience is particularly needed).

The following concerns should be taken into consideration by future academics and developers when creating new authentication schemes for Internet of Things networks and applications, according to this paper’s review of many authentication protocols and schemes:

- Lightweight: As mentioned in Section 2.3.4, sensor-class devices are limited by the availability of memory, processing power and battery capacity. As such, authentication protocols should incur very low computational, communication, and storage costs. Message sizes should remain as small as possible while balancing cost with security, e.g., RFID and smart home applications.
- Security: The robustness of authentication protocols must be carefully examined and taken into account in light of the threat levels posed by a variety of known and potential attacks (such as Sybil attacks, node capture, replay, password guessing, message forgery, brute-force, man-in-the-middle, denial of service, and chosen-plaintext attacks).
- Authentication Efficiency: Real-time, ultra-fast identity authentication is a challenge in several IoT applications, especially in the Internet of Vehicles (IoV). Minimal latency must be considered when designing authentication algorithms to enable high-speed authentication.
- Software/Hardware Integration: Physically unclonable functions can help with identity identification, access control, and traceability, in contrast to purely software-based security techniques. When creating a lightweight, privacy-preserving authentication system for IoT systems, a combination of hardware (greater security) and software (lower cost) solutions should be taken into account.
- Scalability: Extending the dynamic-security update problem described in Section 2.3.4, IoT authentication mechanisms should support scalability to a high number of nodes and admit new nodes without significant reconfigurations, since the IoT is meant to comprise large-scale, heterogeneous networks covering several communication paradigms and application domains; end-to-end identity-authentication mechanisms should support scalability across domains.
- Authentication Mechanism and Hardware Readiness: IoT networks are increasingly vulnerable to hardware flaws as the number of IoT devices rises. It is therefore advisable to implement hardware-level security measures, like tamper-resistant packet processing, before installing IoT devices, since addressing vulnerabilities becomes considerably more difficult after deployment.

## Figures and Tables

**Table 1:** Description of IoT network authentication models.

Authentication Model	Description
Model 1	The user sends the authentication request to the GWN, which forwards the user information to the sensor. The sensor verifies the user information and sends feedback to the GWN, which then authenticates the user.
Model 2	The user sends the authentication request to the GWN, which sends the authentication key to the user while also sending the user information to the sensor. The sensor then authenticates the user.
Model 3	The user sends the authentication request to the GWN, which forwards the user information to the sensor. The sensor then sends its key back to the GWN while authenticating the user.
Model 4	The user sends the authentication request to the sensor, which returns the request to the GWN. The GWN sends a confirmation message to the sensor, which then authenticates the user.
Model 5	The user sends the authentication request to the sensor, which returns the request to the GWN. The GWN authenticates the user and sends a confirmation message to the sensor.

**Table 2:** Evaluation of password-based authentication.

Metric	Analysis
Computational Overhead	Moderate—requires hashing and storage operations.
Energy Efficiency	Low—requires input devices, making it unsuitable for battery-constrained IoT devices.
Scalability	Low—server-side storage increases with the number of users.
Security Resilience	Moderate—vulnerable to brute-force attacks, phishing, and password leaks.

**Table 3:** Evaluation of MAC address-based authentication.

Metric	Analysis
Computational Overhead	Low—simple database lookup.
Energy Efficiency	High—no cryptographic operations required.
Scalability	Moderate—limited by MAC address pool size.
Security Resilience	Low—susceptible to MAC spoofing and replay attacks.

**Table 4:** Evaluation of Public Identity-Based Authentication.

Metric	Analysis
Computational Overhead	High—requires public-key cryptographic operations.
Energy Efficiency	Low—public-key operations are computationally expensive for IoT devices.
Scalability	High—can handle a large number of devices due to distributed nature.
Security Resilience	Moderate—vulnerable to identity spoofing if the key infrastructure is compromised.

**Table 5:** Evaluation of Token-Based Authentication.

Metric	Analysis
Computational Overhead	Moderate—requires storage and token validation.
Energy Efficiency	Moderate—depends on OTP delivery mechanism.
Scalability	High—easily deployable across large IoT networks.
Security Resilience	High—resistant to replay attacks if tokens expire quickly.

**Table 6:** Evaluation of biometric authentication techniques.

Technique	Computational Overhead	Energy Efficiency	Scalability	Security Resilience
Fingerprint Recognition	Moderate—sensor processing	Moderate	High	High—resistant to replay
Facial Recognition	High—deep-learning feature extraction	Low—power-intensive for continuous use	Moderate	Moderate—vulnerable to deepfakes
Iris & Retina Recognition	High—complex image processing	Low	Moderate	High—difficult to forge
EEG-Based Authentication	Very High—neural processing units	Low	Low	Very High—nearly impossible to spoof
PPG-Based Authentication	Moderate—optical sensors	Moderate	High	High—hard to manipulate
Gait Recognition	High—motion sensors + ML	Moderate	High	Moderate—spoofable with synthetic data
Breathing Pattern Recognition	Moderate—respiratory signals	High	Low	High—unique but health-affected
Voice-Based Authentication	Moderate—speech feature extraction	High	High	Moderate—vulnerable to voice spoofing
Multimodal Biometrics	Very High—fusion of features	Low	High	Very High—reduces false acceptance rate

**Table 7:** Comparative summary of IoT authentication mechanisms.

Authentication Method	Computational Overhead	Energy Efficiency	Scalability	Security Resilience	Suitability for IoT
Password-Based	Moderate	Low	Low	Moderate	Not Suitable
MAC-Based	Low	High	Moderate	Low	Not Secure
Public Identity-Based	High	Low	High	Moderate	Resource-Intensive
Token-Based	Moderate	Moderate	High	High	Suitable
Biometric-Based	High	Low	Moderate	Very High	Limited Feasibility
Gait-Based	High	Moderate	High	Moderate	Promising for IoT
Breathing Pattern-Based	Moderate	High	Low	High	Feasible in healthcare IoT
Voice-Based	Moderate	High	High	Moderate	Requires anti-spoofing

**Table 8:** Entropy-based reinterpretation of authentication-class security resilience.

Authentication Class	Typical Credential/Key Space	Approx. Entropy Class	Interpretation
Password-Based	User-chosen alphanumeric string	Low (commonly cited as well under 40 bits for unconstrained human-chosen passwords)	Vulnerable to dictionary and brute-force attacks; effective entropy, not nominal string length, determines resistance.
MAC-Based	48-bit MAC address (not secret; often observable)	Not applicable—identifier is not a secret	Entropy analysis does not apply because the MAC address is transmitted in the clear and can be cloned.
Public Identity-Based	128–256-bit asymmetric key pair	High (128–256 bits of key space)	Brute force infeasible with current classical computing; long-term security depends on key-management hygiene rather than key length alone.
Token-Based (OTP/hardware token)	Time- or counter-synchronized OTP, typically 6–8 decimal digits per use, refreshed frequently	Low per-use nominal entropy, but effective security is provided by short validity windows and non-reuse rather than key length alone	Frequent refresh compensates for the small per-token search space.
Biometric-Based	Continuous physiological/behavioral feature vector	High nominal information content, but usable entropy is reduced by feature-extraction and template-matching tolerance	Practical resistance is better captured by error-rate metrics (Section 3.2.8) than by raw entropy.

**Table 9:** Analysis of authentication schemes in different IoT application domains.

Domain	Authentication Protocol	Cryptographic Technique	Implementation Goal	Attacks to Prevent	Ref.
RFID	Lightweight Mutual Authentication	Physical Unclonable Function (PUF) and lightweight cryptography	Efficient authentication of individual tags	Theft, replay, traceability, cloning, and asynchrony	[58]
RFID	Lightweight Privacy-Preserving Identity Authentication	Ideal PUF environment	Achieve anonymous authentication and forward secrecy	Privacy eavesdropping, asynchrony, imitation, physical attacks, and cloning	[59]
RFID	Lightweight RFID Reader–Writer Mutual Authentication	GNY Logic-Proof Authentication Protocol	Reduce computational and transmission costs	Denial of service, tracking, deception, replay, and eavesdropping	[42]
RFID	Anonymous Ultra-Lightweight NFC Mutual Authentication	Shift and XOR operations	Low computational and storage overhead	Anonymity, tracking, replay, and asynchrony	[60]
Smart Grid	Lightweight Mutual Authentication	Merkle-hash tree	Achieve efficient computation and low communication overhead	Message injection and replay	[43]
Smart Grid	Privacy-Preserving Identity Authentication	HMAC	Achieve low latency in transmission and signature authentication	Device tampering and identity forgery	[61]
Smart Grid	Mutual Authentication and Management	Identity-based encryption for public key management and PKI-based communication	Achieve mutual authentication and key management	Various attacks	[52]
Smart Grid	Lightweight Mutual Authentication	Diffie–Hellman, RSA, AES, and HMAC	Message integrity, low overall communication, and computational overhead	Replay and man-in-the-middle attacks	[37]
Smart Grid	Privacy-Preserving and Gateway-Assisted Identity Authentication	HMAC and homomorphic encryption	Privacy protection, non-repudiation, and traceability	Internal attacks and traffic attacks	[39]
Vehicular IoT	Distributed Aggregate Privacy-Preserving Identity Authentication	Aggregate signature technology	Key management freedom, low message authentication delay, and efficient message processing	Message authentication, conditional linkability, and error information	[62]
Vehicular IoT	Predictive Dual Authentication for Broadcast Identity Authentication	Elliptic curve digital signature algorithm and TESLA	Ensure timely message authenticity and non-repudiation	Data packet loss and memory denial attacks	[63]
Vehicular IoT	Identity Authentication and Re-authentication Scheme	Enhanced dual authentication and key management techniques	Achieve confidentiality and optimal identity authentication delay	Replay attacks, denial of service, location tracking, spoofing, and non-repudiation	[64]
Vehicular IoT	Vehicle Driver Identity Authentication	Elliptic curve cryptography and steganography	Authenticate vehicle driver identity and protect privacy	Information eavesdropping	[65]
Vehicular IoT	Multi-Hop Authentication for Mobile IP	Symmetric polynomial key generation technique	Timely switching without affecting ongoing sessions, reduce potential attacks	Forgery, collusion, replay, man-in-the-middle, and denial of service	[66]
Smart Home	Device Physical Characteristics-Based IoT Security Deployment	Combination of PUF and physical key generation	Security guarantee for tamper evidence	Tampering and un-clonability	[67]
Smart Home	PUF-Based IoT Terminal Device Mutual Authentication Protocol	BAN Logic Proof for Object Lifecycle Correctness	Address confidentiality in the physical-network space-mapping process	Simulation attacks, replay attacks, and eavesdropping attacks	[68]
Smart Home	PUF-Based IoT Device Authentication Scheme	Challenge–response pairs (CRPs) stored in the gateway for mutual authentication between terminal devices and gateways	Identity authentication through the gateway and session key generation for communication	Replay attacks	[68]
Smart Home	Lightweight Mutual Authentication for Real Physical Objects in IoT Environment	AES and CoAP	High computational efficiency, low connection overhead	Resource depletion, denial of service, replay, and physical tampering	[69]
Smart Home	Biometric-Driven Mutual Authentication with One-Time Iris Code	Cryptographic hashing + dynamic iris-code transformation	Sub-2 ms mutual authentication among the user, gateway, and device with formal RoR verification	Impersonation, replay, MITM, offline password guessing, and privileged-insider attacks	[56]

**Table 10:** Representative authentication mechanisms published in 2022–2026, organized by class.

Year	Mechanism/Scheme	Class	Key New Idea	Ref.
**2022**	Lightweight mutual authentication with perfect forward and backward secrecy	Token/Key-based	Session-key freshness guarantees added to a lightweight IoT handshake without heavier public-key operations.	[78]
**2023**	Two-factor lightweight authentication for IoT-enabled healthcare in a quantum-computing setting	Token + Biometric (hybrid)	Combines a lightweight second factor with quantum-computing threat modeling ahead of full PQC adoption.	[79]
**2024**	mWIoTAuth: multi-wearable data-driven implicit IoT authentication	Implicit/Behavioral Biometric	Fuses signals from multiple wearables for continuous, passive authentication rather than a single-sensor biometric.	[37]
**2024/2025**	Systematic reviews of IoT authentication (2014–2023 evidence base)	Cross-class (survey methodology)	Introduce PRISMA-style inclusion/exclusion screening and quantitative performance/security assessment across hundreds of candidate studies.	[12,13]
**2025**	Systematic review of lightweight cryptographic schemes for IoT (77 studies, 2006–2025)	Cross-class (cryptographic primitives)	Applies entropy, execution-time, energy, and NPCR/UACI quantitative metrics uniformly across schemes—the basis for Section 3.2.6, Section 3.2.7, Section 3.2.8 and Section 3.2.9.	[14]
**2026**	Biometric-driven smart-home authentication with a One-Time Iris Code (OTIC)	Biometric	Regenerates a fresh, non-reusable iris-derived code every session using a timestamp and rotation parameter, formally verified with the Real-or-Random model.	[56]

## Data Availability

The data used in this study are available upon request from the corresponding author and are subject to privacy and data-sharing guidelines.
